# Exploring neurophysiological indices in psychiatric disorders: Fronto-central beta oscillations as a potential biomarker

**DOI:** 10.1192/j.eurpsy.2025.2111

**Published:** 2025-08-26

**Authors:** S. Costantini, L. Ghidetti, S. Garofalo, E. Ubaldini, S. Pecar, R. Raggini, M. Beghi, R. P. Sant’Angelo

**Affiliations:** 1 Mental Health, Ausl Romagna; 2 Psychology, University of Bologna; 3 Mental Health Department, Ausl Romagna, Cesena, Italy

## Abstract

**Introduction:**

Historically, psychiatric disorders are studied according to a strictly behavioral approach taking into account the manifest symptomatology.

**Objectives:**

The present work aims to deepen the investigation of neural substrates through the use of neurophysiological recording techniques in an attempt to identify biomarkers related to these pathologies. Investigating whether neurophysiological indices are able to predict the symptomatic improvement and socio-relational functioning of the sample in a transversal manner with respect to the diagnostic category and the symptomatic severity of the psychiatric disorder. Further research is necessary.

**Methods:**

The subjects were evaluated through the scale that measures psychopathological severity (BPRS, PANSS), global functioning (HoNOS) and cognitive (MMSE, CDT). The users were categorized based on the diagnosis and divided into the two Clusters, taking into account the number of hospitalizations of each user during the current year. The following neurophysiological parameters were detected using the Rehacor-T device: electroencephalographic (frontocentral brain oscillations), electrocardiographic (heart rate and HRV) and skin conductance (EDA). For a total procedure duration of 10 minutes (6 minutes of rest, 4 minutes in which the Stroop Test task was performed). Statistical analyses (Random Forest Clustering, Logistic Regression, ANOVA) were performed with the Jasp software (JASP 0.17.3.0).

**Results:**

83 users hospitalized at the Psychiatric Unit of the M. Bufalini hospital in Cesena were recruited and categorized according to the clinical diagnosis. Using a Random Forest Clustering algorithm the population was divided into two different Clusters based on the extent of improvement, assessed based on the low or high number of hospitalizations in the last year. Cluster 1 includes people with a higher number of hospitalizations, while Cluster 2 includes those with a lower number. From logistic regression analyses, a single neuropsychophysiological parameter was identified that was able to predict the classification of users within the two Clusters, namely the Beta oscillations (13-30 Hz) recorded in the fronto-central position in the resting state with eyes closed (resting state). Greater power was recorded in the group of subjects who reported a significant improvement in the symptomatic picture (Cluster 2). The variable of sex was not relevant, while the diagnosis, in Cluster 2, a greater concentration of people with depressive disorder was found.

**Conclusions:**

This work highlights how the fronto-central Beta oscillations recorded in the resting state with eyes closed can be a predictive index of the improvement of the psychopathological conditions of the sample. The remaining neurophysiological indices taken into consideration (delta, theta, alpha and beta oscillations, ECG, EDA), did not show the same predictive capacity.

**Disclosure of Interest:**

None Declared

